# Effect of miglitol on the suppression of nonalcoholic steatohepatitis development and improvement of the gut environment in a rodent model

**DOI:** 10.1007/s00535-017-1331-4

**Published:** 2017-03-27

**Authors:** Yumi Kishida, Hirofumi Okubo, Haruya Ohno, Kenji Oki, Masayasu Yoneda

**Affiliations:** Department of Molecular and Internal Medicine, Graduate School of Biomedical and Health Sciences, Hiroshima University, 1-2-3 Kasumi, Minami-ku, Hiroshima, 734-8551 Japan

**Keywords:** Miglitol, Nonalcoholic steatohepatitis, Inflammation, Glucagon-like peptide 1, Gut microbiota

## Abstract

**Background:**

The gut environment has been considered to play a role in the development of nonalcoholic steatohepatitis (NASH). α-glucosidase inhibitors (α-GIs) delay carbohydrate absorption and may change the gut environment. We considered that the protective effect of α-GIs against NASH development is related to changes in the gut environment and thus investigated the effects of miglitol, an α-GI, on NASH development and the gut environment.

**Methods:**

Mice were divided into three groups and fed a normal chow diet (NCD), a high-fat high-sucrose diet (HFHSD), or HFHSD plus 0.04% miglitol (HFHSD plus M) for 12 weeks.

**Results:**

Insulin resistance developed more in the HFHSD group than in the NCD group, whereas it was suppressed in the HFHSD plus M group. NASH was evaluated histologically, biochemically, and on the basis of messenger RNA expression levels. Miglitol treatment suppressed HFHSD-induced NASH development with the suppression of hepatic Toll-like receptor 4 expression, increased glucagon-like peptide 1 (GLP-1) concentration, and reduced lipopolysaccharide concentration in portal plasma. Regarding the gut environment, the intestinal transit time was shortened and colon inflammation was suppressed in the HFHSD plus M group compared with the HFHSD group. Regarding the gut microbiota, the abundances of *Erysipelotrichaceae* and *Coriobacteriaceae* were increased in the HFHSD group compared with the NCD group, whereas the increase was suppressed in the HFHSD plus M group.

**Conclusions:**

We demonstrated that miglitol has a protective effect against HFHSD-induced NASH development. The increased GLP-1 secretion and the suppression of endotoxemia, associated with the changes in the gut environment, including the gut microbiota, could contribute to the underlying mechanisms.

## Introduction

The prevalence of nonalcoholic fatty liver disease (NAFLD) has been increasing worldwide, along with the increase in the number of people with obesity and metabolic syndrome. Approximately 10–20% of patients with NAFLD are estimated to have nonalcoholic steatohepatitis (NASH), characterized by hepatocellular lipid accumulation along with inflammation and fibrosis [1–3]. Traditionally, the two-hit theory has been accepted as a mechanism of NASH pathogenesis. According to this theory, lipid accumulation in hepatocytes (first hit) is a prerequisite for a second hit, such as oxidative stress, inflammatory cytokines, and endotoxins [4]. This view has been challenged recently by a multiple parallel-hit hypothesis that proposes that multiple hits act together, not independently, in the development of NASH [5]. NASH can lead to cirrhosis and liver cancer; therefore, there is an essential need for therapeutic interventions. However, the current standard treatment of NASH is limited to weight reduction through lifestyle interventions, including dietary modifications and bariatric surgery [6, 7]. α-glucosidase inhibitors (α-GIs) are antidiabetics that delay the absorption of carbohydrates from the small intestine by inhibiting degradation of disaccharides to monosaccharides, thereby providing a postprandial blood glucose lowering effect. Acarbose, voglibose, and miglitol are the α-GIs that are currently used in clinical practice [8]. α-GIs can enhance the production of butyrate (a short-chain fatty acid) in feces [9] and shorten the intestinal transit time in humans [10]. These findings suggest that α-GIs can change the gut environment. On the other hand, there are studies reporting a prolonged intestinal transit time, increased intestinal permeability, and altered gut microbiota in patients with NAFLD/NASH, which may indicate changes in their gut environment [11–13]. In addition, α-glucosidase activity reportedly increased in the intestinal tract in high-fat-diet-induced obese rats [14]. It has also been reported that acarbose showed a protective effect against NASH development in high-fat-induced obese rats [15]. However, the mechanisms of these effects have not been fully elucidated. We considered that the protective effects of α-GIs against NASH development were related to changes in the gut environment. Thus, we investigated the effects of miglitol on NASH development and the gut environment using a high-fat high-sucrose diet (HFHSD)-induced NASH model mice.

## Methods

### Animals and treatments

Five-week-old male C57BL/6 mice were purchased from Charles River Japan (Kanagawa, Japan). The mice were housed in temperature- and light-controlled rooms with free access to food and water. At 6 weeks of age, the mice were divided into three groups (*n* = 10 per group) and fed, respectively, a normal chow diet (NCD; 12.7 kcal% fat, 61.6 kcal% carbohydrate, and 25.7 kcal% protein), HFHSD (44.6 kcal% fat, 40.6 kcal% carbohydrate, and 14.8 kcal% protein), or HFHSD containing 0.04% miglitol (HFHSD plus M) for 12 weeks. The HFHSD was prepared by Oriental Yeast (Tokyo, Japan) according to the method described in a previous study [16]. Miglitol was supplied by Sanwa Kagaku Kenkyusho (Aichi, Japan). The mice were killed after having fasted for 6 h; portal blood, the liver, and the colon were then collected, and the fecal contents of the entire large intestine were weighed. The mice were handled in accordance with the guidelines for the care and use of experimental animals published by the Japanese Association for Laboratory Animal Science, and animal experiments were performed in strict accordance with the recommendations in the Guide for the Care and Use of Laboratory Animals of the Hiroshima University Animal Research Committee. All protocols were approved by the Institutional Review Board of Hiroshima University.

### Histochemical studies

Paraffin-embedded liver and colon sections were stained with hematoxylin–eosin (HE), Sirius red, and an antibody for α-smooth muscle actin at Kyodo Byori (Kobe, Japan). Images were obtained with a multifunctional microscope (BZ-9000; Keyence, Osaka, Japan). Evaluation of disease progression was performed according to the histological scoring system for NAFLD [17], and the number of positively stained areas was counted in 12 randomly selected fields (×20 or ×40) and analyzed with use of the BZ-9000 microscope.

### Biochemical analysis

The blood glucose level was measured with a Medisafe mini system (Terumo, Tokyo, Japan). The plasma insulin level was measured with a mouse insulin ELISA kit (Sibayagi, Gunma, Japan). Homeostatic model assessment insulin resistance (HOMA-IR) was calculated as fasting insulin (mU/L) × fasting glucose (mmol/L)/22.5 [18]. Hepatic lipids were extracted and assayed by the Folch method [19]. The hepatic triglyceride content was assayed with the triglyceride E test (Wako, Osaka, Japan). The plasma alanine aminotransferase level was measured with a transaminase C-II test kit (Wako, Osaka, Japan). The plasma active glucagon-like peptide 1 (GLP-1) concentration was measured with a mouse active GLP-1 ELISA kit (Shibayagi, Gunma, Japan). The plasma lipopolysaccharide-binding protein (LBP) concentration was measured with an enzyme immunoassay for the determination of mouse LBP (Biometec, Greifswald, Germany). The plasma lipopolysaccharide (LPS) concentration was measured with a mouse LPS ELISA kit (Cusabio, Wuhan, China).

### Quantitative real-time reverse transcription PCR

Total RNA was extracted from mouse livers and colons with use of TRIzol (Invitrogen, Carlsbad, CA, USA). First-strand DNA was synthesized with PrimeScript RT master mix (Takara, Kyoto, Japan). Quantitative real-time reverse transcription PCR was performed with SYBR Green master mix (Takara) and an ABI 7500 real-time PCR system (Applied Biosystems, Foster City, CA, USA). The messenger RNA (mRNA) gene expression levels were normalized to that of the glyceraldehyde 3-phosphate dehydrogenase gene, and relative expression was determined by the comparative Ct method. The primers, designed as previously reported [20–24], were as follows: glyceraldehyde 3-phosphate dehydrogenase forward TGATGGGTGTGAACCACGAG, reverse GGGCCATCCACAGTCTTCTG; fatty acid synthase forward GCTGCGGAAACTTCAGGAAAT, reverse AGAGACGTGTCACTCCTGGACTT; carnitine palmitoyltransferase 1 forward CCAGGCTACAGTGGGACATT, reverse GAACTTGCCCATGTCCTTGT; CD36 forward TGCTGGAGCTGTTATTGGTG, reverse TGGGTTTTGCACATCAAAGA; tumor necrosis factor α forward GTAGCCCACGTCGTAGCAAAC, reverse CTGGCACCACTAGTTGGTTGTC; IL-1β forward TGGGCCTCAAAGGAAAGAAT, reverse CTTGGGATCCACACTCTCCA; transforming growth factor β forward ATTCCTGGCGTTACCTTG, reverse CTGTATTCCGTCTCCTTGGTT; Toll-like receptor 4 (TLR4) forward GCCTTTCAGGGAATTAAGCTCC, reverse AGATCAACCGATGGACGTGTAA; CD14 forward GAGTTGTGACTGGCCCAGTCAGC, reverse GCAAAAGCCAGAGTTCCTGAC; IL-6 forward CCATCCAGTTGCCTTCTTGG, reverse TCCACGATTTCCCAGAGAACA.

### Intestinal transit time

Carmine red (10 mg per liter of water, 10 μL per gram of body weight) was administered orally to each mouse. The total intestinal transit time was defined as the time between the ingestion of carmine red and the first appearance of colored feces.

### Gut microbiota analyses by next-generation sequencing

Fresh feces were collected immediately after defecation, frozen, and stored for gut microbiota analyses after 12 weeks of the diet. Total DNA was extracted from frozen feces with use of a QIAamp DNA stool mini Kit (Qiagen, Valencia, CA, USA) as previously described [25]. For each sample, the V3–V4 region of the 16S ribosomal RNA (rRNA) gene was amplified with use of primer pairs 341F (5′-TCGTCGGCAGCGTCAGATGTGTATAAGAGACAGCCTACGGGAGGCAGCAG-3′) and 785R (5′-GTCTCGTGGGCTCGGAGATGTGTATAAGAGACAGGACTACCAGGGTATCTAATCC-3′), with Illumina adapter overhang nucleotide sequences as indicated by the underlines. Amplicons were purified, indexed, and sequenced according to the “16S Metagenomic Sequencing Library Preparation” protocol. We used the software program QIIME (version 1.8.0) [26] to analyze the 16S rRNA sequence generated from paired-end amplicon sequencing. Paired Illumina reads were assembled with QIIME’s fastq-join. All reads from paired end sequencing were quality filtered with use of QIIME’s script split_libraries_fastq.py. Chimera detection was performed with USEARCH version 6.1 [27] against the 97% clustered representative sequences from GreenGenes version 13.8 [28]. Operational taxonomic unit data were generated with pick_open_reference_otus.py with use of the default uclust method and GreenGenes version 13.8. Subsequent analyses of diversity were performed at a depth of 10,000 sequences per sample. Alpha diversity was assessed by the Shannon index. Beta diversity was studied by a principal coordinates analysis, measuring dissimilarities at phylogenetic distance based on weighted Unifrac analysis. We also used QIIME to estimate the relative abundance of bacterial groups at different taxonomic levels between each group of mice.

### Statistical analysis

Results are expressed as the mean ± standard error of the mean. Statistical significance was assessed by ANOVA followed by the Tukey honestly significant difference test. Statistical analyses were performed with IBM SPSS Statistics version 22.0 (IBM, Armonk, NY, USA) or R (version 3.1.3; R Foundation for Statistical Computing, Vienna, Austria). We took *p* < 0.05 to indicate a statistically significant difference.

## Results

### Miglitol treatment suppressed HFHSD-induced NASH development

**Fig. 1:**
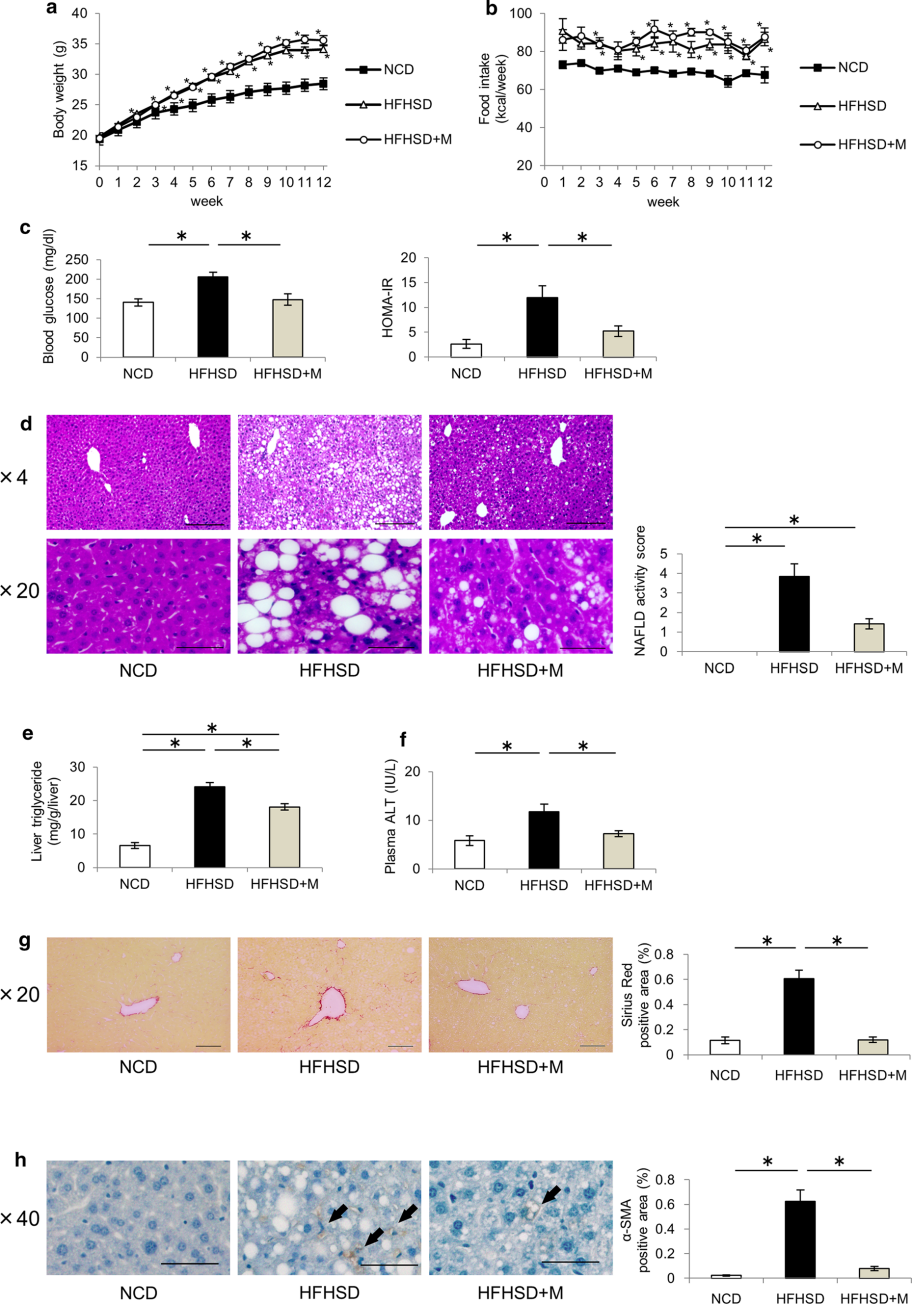
Miglitol treatment suppressed high-fat high-sucrose diet (*HFHSD*)-induced nonalcoholic steatohepatitis development. Mice were divided into three groups and fed a normal chow diet (*NCD*), HFHSD, or HFHSD containing 0.04% miglitol (*HFHSD+M*) for 12 weeks and then killed. **a** Weekly body weight changes (*n* = 10 per group). **b** Weekly food intake changes (*n* = 10 per group). **c** Fasting blood glucose levels (*n* = 9–10 per group) and homeostatic model assessment insulin resistance (*HOMA-IR*; *n* = 6–9 per group). **d** Liver sections were stained with hematoxylin–eosin. *Scale bar* 200 μm (×4 magnification) or 50 μm (×20 magnification). Nonalcoholic fatty liver disease (*NAFLD*) activity scores were determined for each mouse. **e** Hepatic triglyceride levels (*n* = 6–7 per group). **f** Plasma alanine aminotransferase (*ALT*) levels (*n* = 6–7 per group). **g** Liver sections were stained with Sirius red. *Scale bar* 100 μm (×20 magnification). Positively stained areas were counted with use of a Keyence BZ-9000 microscope. **h** Liver sections were stained with α-smooth muscle actin (*α-SMA*). *Scale bar* 50 μm (×40 magnification). *Arrows* indicate α-SMA-positive areas. Positively stained areas were counted with use of a Keyence BZ-9000 microscope. Data are presented as the mean ± standard error of the mean. *Asterisk* statistical significance (*p* < 0.05)

To evaluate the protective effects of miglitol against NASH development, the mice were fed the NCD, HFHSD, or HFHSD plus M for 12 weeks. The HFHSD group showed increased body weight and food intake compared with the NCD group, but there were no significant differences between the HFHSD group and the HFHSD plus M group (Fig. 1a, b). The HFHSD group showed increased levels of fasting blood glucose and HOMA-IR compared with the NCD group, whereas these changes were suppressed in the HFHSD plus M group (Fig. 1c). The livers were harvested and subjected to histological analysis. HE staining of the livers from the HFHSD group showed accumulation of lipid droplets, inflammatory cell infiltration, and an increase in the numbers of ballooning hepatocytes compared with the NCD group, whereas these changes were suppressed in the HFHSD plus M group (Fig. 1d). Biochemical analysis revealed the hepatic triglyceride content and plasma alanine aminotransferase levels to be higher in the HFHSD group than in the NCD group, whereas these changes were suppressed in the HFHSD plus M group (Fig. 1e, f). Sirius red staining was performed to evaluate fibrotic changes in the liver. The number of positively stained areas increased in the HFHSD group compared with the NCD group, whereas this increase was suppressed in the HFHSD plus M group (Fig. 1g). Immunostaining with α-smooth muscle actin, a marker of the activation of stellate cells, which plays a role in liver fibrosis, showed that the number of positively stained areas increased in the HFHSD group compared with the NCD group, whereas this increase was suppressed in the HFHSD plus M group (Fig. 1h). These results showed that miglitol had protective effects against hepatocellular lipid accumulation, inflammation, and fibrosis, thereby suppressing NASH development.

### Miglitol treatment suppressed the expression of genes involved in NASH pathogenesis

**Fig. 2:**
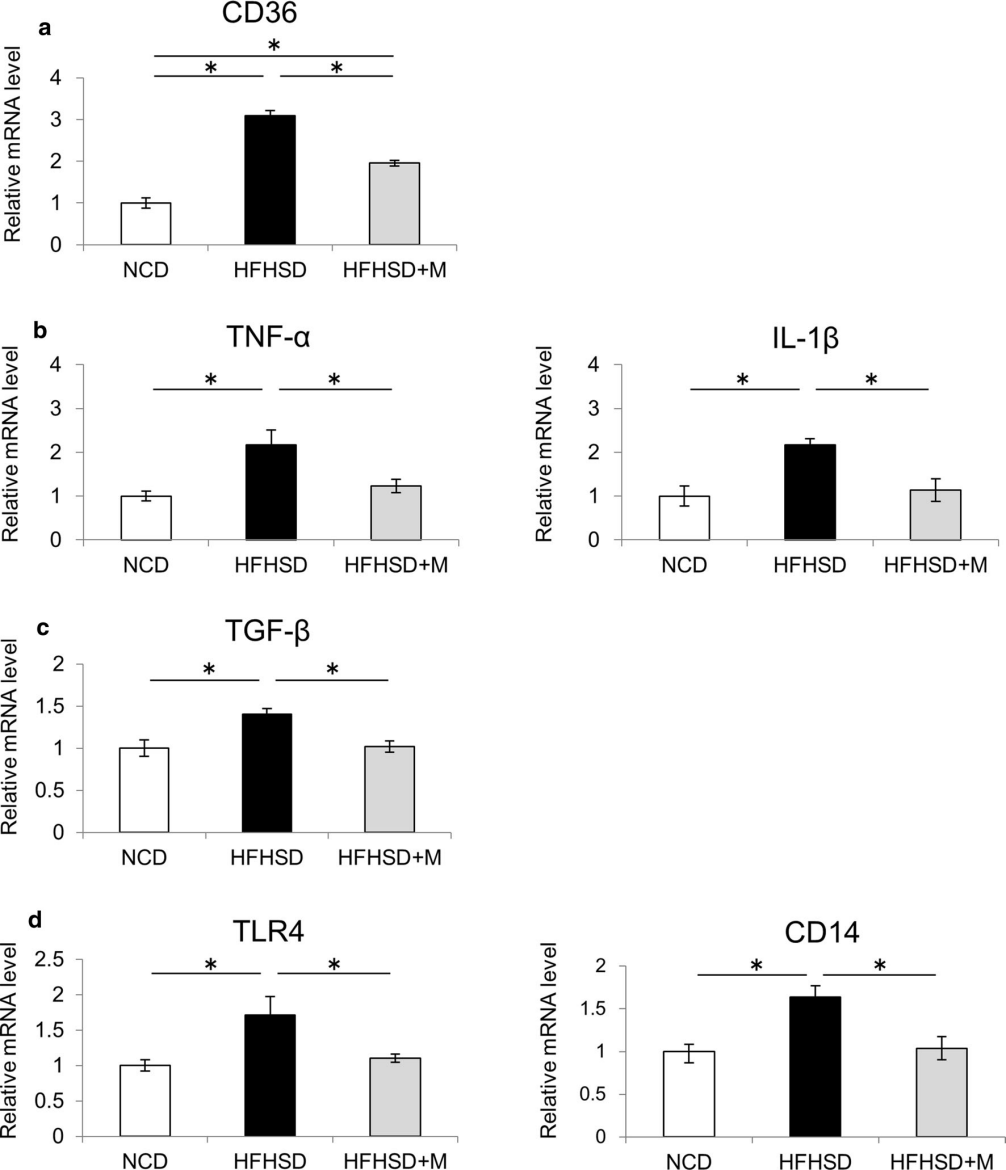
Miglitol treatment suppressed the expression of genes involved in nonalcoholic steatohepatitis pathogenesis. Mice were divided into three groups and fed a normal chow diet (*NCD*), a high-fat high-sucrose diet (*HFHSD*), or HFHSD containing 0.04% miglitol (*HFHSD+M*) for 12 weeks and then killed. **a** Hepatic messenger RNA (*mRNA*) levels of CD36 (*n* = 5–7 per group). **b** Hepatic mRNA levels of tumor necrosis factor α (*TNF-α*) and IL-1β (*n* = 5–7 per group). **c** Hepatic mRNA levels of transforming growth factor β (*TGF-β*) (*n* = 5–7 per group). **d** Hepatic mRNA levels of Toll-like receptor 4 (*TLR4*) and CD14 (*n* = 5–7 per group). Data are presented as the mean ± standard deviation. *Asterisk* statistical significance (*p* < 0.05)

To elucidate the mechanism of the protective effects of miglitol against NASH development, the gene expression levels of factors related to lipid accumulation, inflammation, and fibrosis in the liver were analyzed. The mRNA levels of fatty acid synthase (involved in fatty acid synthesis) and carnitine palmitoyltransferase 1 (involved in β-oxidation) were not significantly different among the three groups (data not shown). The mRNA level of CD36 (involved in fatty acid uptake) increased in the HFHSD group compared with the NCD group, whereas this increase was suppressed in the HFHSD plus M group (Fig. 2a). A previous study showed that CD36 expression in the liver was increased by inflammatory cytokines [29]. On the basis of this, we examined the expression of inflammatory cytokines in the liver. The mRNA levels of tumor necrosis factor α and IL-1β in the liver were increased in the HFHSD group compared with the NCD group, whereas these changes were suppressed in the HFHSD plus M group (Fig. 2b). Subsequently, transforming growth factor β, a marker of hepatic fibrosis, was analyzed. The mRNA level of transforming growth factor β in the liver was increased in the HFHSD group compared with the NCD group, whereas the increase was suppressed in the HFHSD plus M group (Fig. 2c). Finally, expression of TLR4 and CD14 (a coreceptor for TLR4) in the liver was analyzed. The mRNA levels of TLR4 and CD14 in the liver were increased in the HFHSD group compared with the NCD group, whereas these changes were suppressed in the HFHSD plus M group (Fig. 2d).

### Miglitol changed the gut environment and reduced endotoxemia

**Fig. 3:**
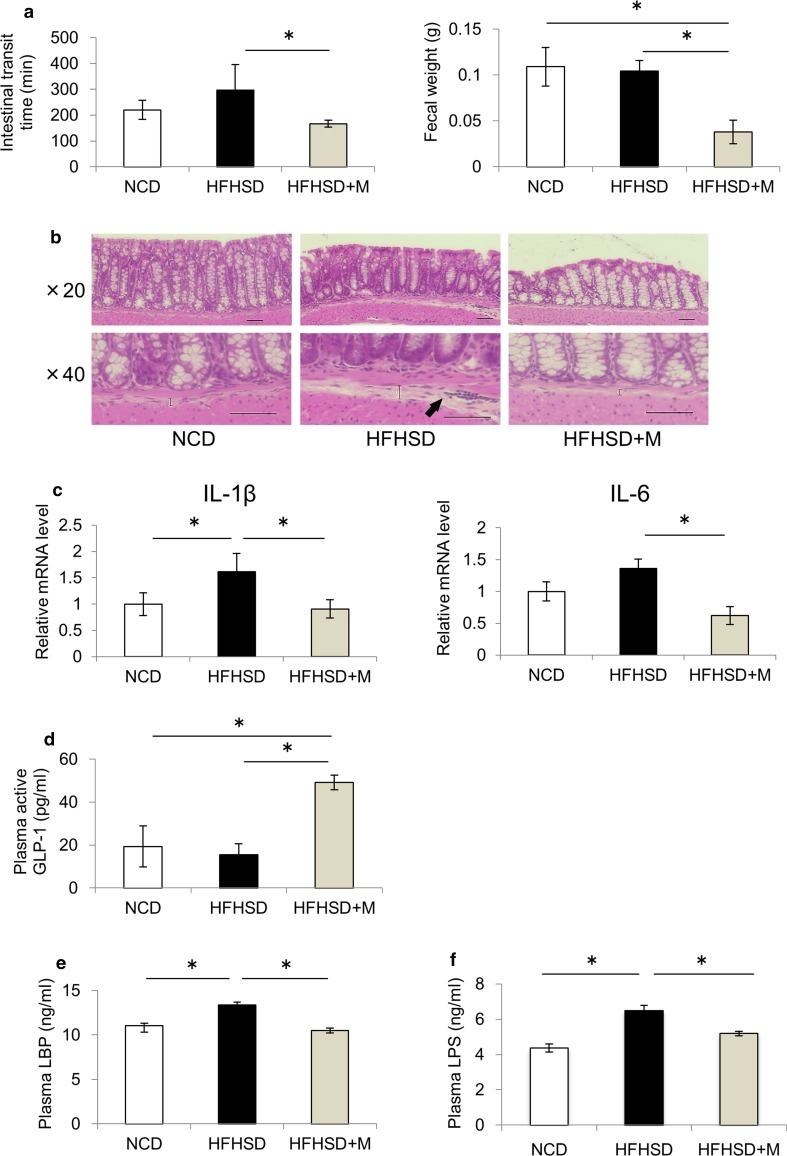
Miglitol changed the gut environment and reduced endotoxemia. Mice were divided into three groups and fed a normal chow diet (*NCD*), a high-fat high-sucrose diet (*HFHSD*), or HFHSD containing 0.04% miglitol (*HFHSD+M*) for 12 weeks and then killed. **a** The intestinal transit times were determined by measurement of the time elapsed and the color of excreted feces. The colonic fecal weight was determined at the time when the mice were killed (*n* = 10 per group). **b** Colon sections were stained with hematoxylin–eosin. *Scale bar* 50 μm (×20, ×40 magnification). The *straight line* indicates the extent of the submucosal edema and *arrows* indicate inflammatory infiltrates composed primarily of neutrophils. **c** Colonic messenger RNA (*mRNA*) levels of IL-1β and IL-6 (*n* = 4–5 per group). **d** Plasma active glucagon-like peptide 1 (*GLP-1*) levels (*n* = 6–8 per group). **e** Plasma lipopolysaccharide-binding protein (*LBP*) levels (*n* = 6–8 per group). **f** Plasma lipopolysaccharide (*LPS*) levels (*n* = 6–7 per group). Data are presented as the mean ± standard error of the mean. *Asterisk* statistical significance (*p* < 0.05)

Miglitol acts on the intestine; thus, we examined the effect of miglitol on the gut environment. First, we examined the effect of miglitol on the intestinal transit time. Although there was no significant difference in the intestinal transit time between the NCD group and the HFHSD group, the administration of miglitol shortened the intestinal transit time and reduced the amount of residual feces in the colon (Fig. 3a). Inflammatory changes in the colon were also examined. HE staining of the colon showed that signs of early inflammation, as evidenced by submucosal edema and inflammatory cell infiltration, were present in the HFHSD group, whereas these changes were suppressed in the HFHSD plus M group (Fig. 3b). The mRNA levels of IL-1β and IL-6 in the colon also increased in the HFHSD group compared with the NCD group, whereas these changes were suppressed in the HFHSD plus M group (Fig. 3c). Subsequently, portal plasma concentrations of GLP-1, an intestinal hormone, were measured. The plasma GLP-1 concentrations were higher in the HFHSD plus M group than in the HFHSD group (Fig. 3d). Finally, TLR4-signaling-related substances were examined in the portal blood since there was a change in TLR4 expression in the liver. The portal plasma concentrations of LBP (a protein required to transfer LPS to CD14) and LPS were increased in the HFHSD group compared with the NCD group, whereas this increase was not observed in the HFHSD plus M group (Fig. 3e, f). These results suggest that miglitol suppressed colon inflammation, prevented endotoxin influx from the intestinal tract to the liver, and downregulated TLR4 signaling in the liver.

### Gut microbiota changes in response to miglitol treatment

**Fig. 4:**
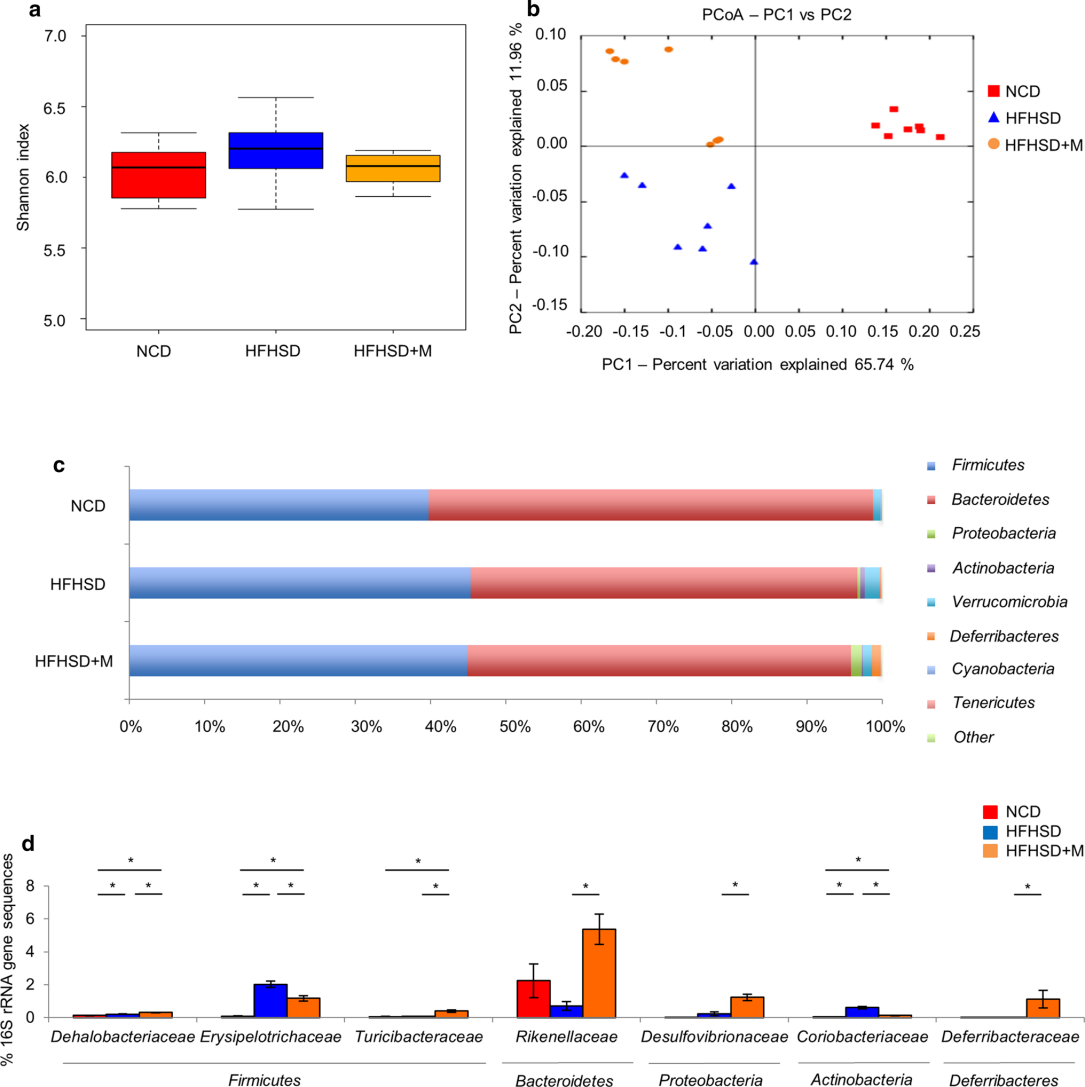
Gut microbiota changes in response to miglitol treatments. Mice were divided into three groups and fed a normal chow diet (*NCD*), a high-fat high-sucrose diet (*HFHSD*), or HFHSD containing 0.04% miglitol (*HFHSD+M*) for 12 weeks and then killed. **a** Estimates of bacterial diversity as assessed by the Shannon index (*n* = 7 per group). **b** Principal coordinates analysis (*PCoA*) plots based on the weighted Unifrac distance matrices showing the clustering of global microbiota (*n* = 7 per group). **c** Relative abundance of phyla in fecal samples between groups of mice (*n* = 7 per group). **d** Relative abundance of families in fecal samples between groups of mice (*n* = 7 per group). Data are presented as the mean ± standard error of the mean. *PC* principal coordinate, *rRNA* ribosomal RNA, *asterisk* statistical significance (*p* < 0.05)

The effects of miglitol on the gut microbiota were examined by 16S rRNA gene sequencing. The Shannon index was used to evaluate the diversity of gut microbiota, but there was no significant difference in the biodiversity among the groups (Fig. 4a). Then, principal coordinates analysis was used to compare the gut microbial communities among the groups. The samples representing the same group were clustered together, and those representing different groups were clustered separately (Fig. 4b). An analysis of similarity test showed significant differences among the groups, suggesting that the gut microbial communities were different among the three groups. The differences in the gut microbial communities were further examined according to bacterial classification. The percentage of *Bacteroidetes* was lower in the HFHSD group than in the NCD group, whereas there was no significant difference between the HFHSD group and the HFHSD plus M group. The percentage of the *Actinobacteria* was higher in the HFHSD group than in the NCD group, whereas the increase was suppressed in the HFHSD plus M group (Fig. 4c). Subsequently, the families of the bacteria showing a different distribution between the HFHSD group and the HFHSD plus M group were identified. As a result, the percentages of the 16S rRNA gene sequences representing the *Erysipelotrichaceae* within the phylum *Firmicutes* and the *Coriobacteriaceae* within the phylum *Actinobacteria* were higher in the HFHSD group than in the NCD group, whereas the increases were suppressed in the HFHSD plus M group (Fig. 4d). These results suggested that miglitol significantly changed the intestinal microbial communities.

## Discussion

This study demonstrates that miglitol suppressed hepatocellular lipid accumulation, inflammation, and fibrosis, shortened the intestinal transit time, suppressed inflammation, and changed the microbiota in the colons of HFHSD-induced NASH mice. These protective effects of miglitol on the liver and colon might be mediated by the inhibition of endotoxin influx into the portal blood and by an increase of plasma GLP-1 concentrations.

Miglitol did not reduce the body weight but reduced insulin resistance (Fig. 1a, c). Insulin resistance is thought to play an important role in the development of NASH [30]. Previous studies have reported that miglitol reduced insulin resistance in drug-naive metabolic syndrome patients [31] and in high-fat-diet-induced obese mice [32]. These studies also reported a reduction of body weight. In contrast, in the present study, miglitol did not reduce the body weight but reduced HOMA-IR. Therefore, we consider that a factor independent of body weight might be involved in the mechanism of reduction of insulin resistance following miglitol administration.

Endotoxin-driven TLR4 signaling is thought to be related to insulin resistance. Several studies reported that insulin resistance and hepatic steatosis were suppressed in TLR4 and CD14 mutant mice [33, 34]. Endotoxin LPS influx from the intestinal tract to the portal vein due to enhanced intestinal permeability may induce the expression of inflammatory cytokines through TLR4 in the liver, and thereby play an important role in the development of NASH [35, 36]. In the present study, we showed that the expression levels of TLR4 in the liver as well as plasma LPS concentrations were increased in the HFHSD group compared with the NCD group, whereas these changes were suppressed in the HFHSD plus M group (Figs. 2d, 3f). These data suggest that the expression levels of TLR4 in the liver were increased by the HFHSD-induced influx of LPS into the portal vein, and miglitol treatment suppressed this process. Thus, protection from NASH development by miglitol treatment appears to be at least partially attributable to the suppression of endotoxin-driven TLR4 signaling.

As shown in Fig. 3, the maintenance of intestinal barrier function through suppression of colon inflammation is considered to be one of miglitol’s protective mechanisms of action against endotoxemia. It has been reported that administration of an α-GI increased the levels of butyric acid in the intestines of healthy individuals [9]. In addition, another report demonstrated that administration of butyric acid suppressed intestinal inflammation in mice [37]. These findings suggest an association between the increase in the butyric acid level in the intestine caused by miglitol and the suppression of colon inflammation. On the other hand, it has been reported that an α-GI shortened the intestinal transit time in humans [10]. In the present study, miglitol shortened the intestinal transit time and reduced the amount of residual feces in the intestines. We have previously reported that administration of a gastroprokinetic agent reduced the amount of residual feces, changed the gut microbiota, and suppressed colon inflammation in a mouse model [23], which suggested that the effect could also contribute to suppression of colon inflammation.

In the present study, the portal plasma GLP-1 concentrations were higher in the HFHSD plus M group than in the HFHSD group (Fig. 3d). GLP-1 acts as a suppressor of intestinal inflammation via intestinal intraepithelial lymphocytes [38]. It has been reported that administration of exenatide, an analog of GLP-1, suppressed the hepatocellular lipid accumulation and inflammation and suppressed NASH development in a mouse model [39]. Therefore, we considered that the increase in plasma GLP-1 concentrations induced by miglitol might have contributed to the suppression of NASH development by maintaining intestinal barrier function through the suppression of intestinal inflammation as described above and through direct effects on the liver. In addition, several studies have reported that miglitol increased the blood GLP-1 concentrations in patients with type 2 diabetes, whereas other α-GIs did not [40, 41]. An underlying mechanism of this effect is that unlike other α-GIs, miglitol is absorbed in the upper part of the small intestine and can effectively stimulate GLP-1 secretion, whereas most carbohydrates are absorbed in the lower part of the small intestine, where GLP-1-producing L cells are located [40, 41]. Therefore, we consider that the increase in the blood GLP-1 concentrations is an important effect of miglitol in the suppression of NASH development as compared with other α-GIs.

Regarding the composition of intestinal bacteria, it was reported that acarbose increased the populations of *Bifidobacterium longum* in Chinese patients with type 2 diabetes mellitus [42], which was not observed in our study, perhaps owing to the differences between the species, drugs, and methods of analysis. In this study, the percentages of the 16S rRNA gene sequences representing the family *Erysipelotrichaceae* within the phylum *Firmicutes* and the family *Coriobacteriaceae* within the phylum *Actinobacteria* were higher in the HFHSD group than in the NCD group, whereas the increases were suppressed in the HFHSD plus M group (Fig. 4d). Increased percentages of *Erysipelotrichaceae* have been reported in mice maintained on a Western-style diet and in enteritis model mice, suggesting an association of the gut microbiota with the diet and enteritis [43] In addition, an association between *Coriobacteriaceae* and hepatic triglyceride accumulation has been reported [44]. Therefore, we consider that the changes in the gut microbiota are related to the suppression of intestinal inflammation and NASH development.

In conclusion, we demonstrated a protective effect of miglitol against NASH development. The drug changed the gut environment, including the gut microbiota, and increased the GLP-1 secretion in the portal vein and suppressed endotoxemia. Our results suggest that miglitol can suppress the development of NASH by improving the gut environment.

## Abbreviations

α-GI: α-glucosidase inhibitor
GLP-1: Glucagon-like peptide 1
HE: Hematoxylin–eosin
HFHSD: High-fat high-sucrose diet
HOMA-IR: Homeostatic model assessment insulin resistance
LBP: Lipopolysaccharide-binding protein
LPS: Lipopolysaccharide
mRNA: Messenger RNA
NAFLD: Nonalcoholic fatty liver disease
NASH: Nonalcoholic steatohepatitis
NCD: Normal chow diet
rRNA: Ribosomal RNA
TLR4: Toll-like receptor 4
